# Tumor-derived exosomal ADAM17 promotes pre-metastatic niche formation by enhancing vascular permeability in colorectal cancer

**DOI:** 10.1186/s13046-024-02991-3

**Published:** 2024-02-27

**Authors:** Keyu Li, Wenhua Xue, Zhihua Lu, Suo Wang, Jiayao Zheng, Kuangyi Lu, Ming Li, Yang Zong, Feng Xu, Jiamin Dai, Yang Yang, Jinbing Sun

**Affiliations:** 1 Department of General Surgery, Changshu No. 1 People’s Hospital, Affiliated Changshu Hospital of Soochow University, No. 1 Shuyuan Street, Changshu, 215500 Jiangsu China; 2 Department of Pharmacy, The First Affiliated Hospital of Zhengzhou University, Zhengzhou, 450052 Henan P.R. China; 3 Affiliated Hospital of Integrated Traditional Chinese and Western Medicine, Nanjing University of Chinese Medicine, Nanjing, 210028 Jiangsu China; 4 Department of Radiology, Dushu Lake Hospital Affiliated to Soochow University, Medical Center of Soochow University, Suzhou Dushu Lake Hospital, Suzhou, 215123 Jiangsu China

**Keywords:** Exosome, ADAM17, Colorectal cancer, Hematogenous metastasis, VE-cadherin

## Abstract

**Background:**

Hematological metastasis has been recognized as a crucial factor contributing to the high rates of metastasis and mortality observed in colorectal cancer (CRC). Notably, exosomes derived from cancer cells participate in the formation of CRC pre-metastatic niches; however, the mechanisms underlying their effects are largely unknown. While our preliminary research revealed the role of exosome-derived disintegrin and metalloproteinase 17 (ADAM17) in the early stages of CRC metastasis, the role of exosomal ADAM17 in CRC hematogenous metastasis remains unclear.

**Methods:**

In the present study, we isolated and purified exosomes using ultracentrifugation and identified exosomal proteins through quantitative mass spectrometry. In vitro, co-culture assays were conducted to evaluate the impact of exosomal ADAM17 on the permeability of the blood vessel endothelium. Vascular endothelial cell resistance, the cell index, membrane protein separation, flow cytometry, and immunofluorescence were employed to investigate the mechanisms underlying exosomal ADAM17-induced vascular permeability. Additionally, a mouse model was established to elucidate the role of exosomal ADAM17 in the modulation of blood vessel permeability and pre-metastatic niche formation in vivo.

**Results:**

Our clinical data indicated that ADAM17 derived from the circulating exosomes of patients with CRC could serve as a blood-based biomarker for predicting metastasis. The CRC-derived exosomal ADAM17 targeted vascular endothelial cells, thus enhancing vascular permeability by influencing vascular endothelial cadherin cell membrane localization. Moreover, exosomal ADAM17 mediated the formation of a pre-metastatic niche in nude mice by inducing vascular leakage, thereby promoting CRC metastasis. Nonetheless, ADAM17 selective inhibitors effectively reduced CRC metastasis in vivo.

**Conclusions:**

Our results suggest that exosomal ADAM17 plays a pivotal role in the hematogenous metastasis of CRC. Thus, this protein may serve as a valuable blood-based biomarker and potential drug target for CRC metastasis intervention.

**Supplementary Information:**

The online version contains supplementary material available at 10.1186/s13046-024-02991-3.

## Background

Tumor metastasis is the primary cause of death among patients with colorectal cancer (CRC) [1]. Notably, metastasis is a complex multistep process involving invasion into surrounding tissue at the primary site, intravasation, circulation in the bloodstream, and extravasation of tumor cells to distant organs [2, 3]. Throughout the pre-metastatic phase, changes in vascular permeability emerge as a crucial factor in the hematogenous metastasis of CRC, wherein the heightened invasiveness of tumor cells is closely linked to the increased permeability of the vascular endothelial cell barrier [3–5]. Notably, various biomarkers associated with CRC hematogenous metastasis have proven valuable in cancer diagnosis and prognosis prediction [4, 6, 7]. Furthermore, targeting vascular permeability may represent a promising strategy for disrupting the pre-metastatic niche formation [8–10]. Therefore, the identification of biomarkers and drug targets associated with pre-metastatic niche formation, particularly in the context of hematogenous metastasis in CRC, holds significant value for the diagnosis of CRC and the development of drugs to treat CRC metastasis.

Exosomes in the serum of patients with CRC can mediate various stages of tumor development, invasion, metastasis, and drug resistance; notably, exosomes released by metastatic tumor cells play an important role in facilitating tumor invasion and metastasis [11]. Tumor-derived exosomes carry proteins, lipids, and various nuclear acids that promote increased permeability of the vascular endothelial barrier, ultimately inducing CRC hematogenous metastasis and pre-metastatic niche formation [12]. Our preliminary clinical studies revealed elevated levels of exosome-derived A disintegrin and metalloproteinase 17 (ADAM17) in the serum of patients with metastatic CRC. ADAM17, also known as the tumor necrosis factor-alpha converting enzyme, is a membrane protein belonging to the ADAM protein family [13]. Notably, the expression of this protein is significantly higher in metastatic than in non-metastatic patients and normal individuals [14]. Additionally, ADAM17 exhibits high expression in various tumor types, influencing tumor progression [15]. Mechanistically, our preliminary findings suggest that exosomal ADAM17 effectively enhances the migratory capacity of CRC cells by cleaving the E-cadherin junctions [14]. However, the precise molecular mechanisms underlying the role of epithelial–mesenchymal transition (EMT) CRC cell-derived exosomal ADAM17 in the hematogenous metastasis of CRC remain unclear.

The clinical data analyzed in the present study indicated that ADAM17 derived from circulating exosomes within the serum of patients with CRC could be employed as a blood-based biomarker to predict metastasis. Furthermore, these results revealed that CRC-derived exosomal ADAM17 targets vascular endothelial cells, thereby promoting vascular permeability by influencing vascular endothelial (VE)-cadherin cell membrane localization level. Furthermore, we demonstrated that exosomal ADAM17 mediates the formation of a pre-metastatic niche in nude mice by inducing vascular leakage, thereby promoting CRC metastasis. Finally, ADAM17 selective inhibitors significantly reduced CRC metastasis in vivo; therefore, these ADAM17 inhibitors are promising therapeutic candidates for further development, holding the potential to effectively inhibit CRC metastasis in clinical practice.

## Methods

### Specimen collection

Human peripheral blood samples, tumor tissues, and adjacent tissue specimens were obtained from patients with CRC at the Department of General Surgery of Changshu No. 1 People’s Hospital affiliated with Soochow University; patients receiving neoadjuvant chemotherapy/radiotherapy were excluded. CRC staging was determined using the modified 2003 TNM staging system provided by The International Union Against Cancer/American Joint Committee on Cancer. Patient groups were matched for age and sex, and all cases were pathologically confirmed (Table 1). Informed consent was obtained from all participants for blood donation, adhering to approved institutional protocols. Blood samples were collected in tubes containing ethylenediaminetetraacetic acid (EDTA) and centrifuged at 2500 × *g* for 10 min to extract serum for further analysis [14]. Plasma exosomes were isolated according to the procedure described below. All procedures involving human participants were performed in accordance with the ethical standards set by the institutional and/or national research committee, following the 1964 Helsinki Declaration and its subsequent amendments and comparable ethical standards. This study was approved by the Ethics Committee of Changshu No. 1 People’s Hospital Affiliated with Soochow University. Informed consent was obtained from all study participants [14, 16].

**Table 1:** Relationship between the serum CirExo-ADAM17 levels and clinicopathological features of patients with colorectal cancer (CRC).

Parameter	Number of patients		CirExo-ADAM17 level		χ2	P*-*value
	Number	%	<median	≥median		
**Gender**						
Male	40	50	17	23	1.251	0.2634
Female	40	50	22	18		
**Age, years**						
< 60	38	47.5	19	19	0.04527	0.8315
≥ 60	42	52.5	20	22		
**Tumor site**						
Colon	38	47.5	21	17	1.229	0.2676
Rectal	42	52.5	18	24		
**Tumor size, cm**						
< 5	43	53.75	25	18	3.281	0.0701
≥ 5	37	46.25	14	23		
**Tumor grade**						
Moderate/well	39	48.75	23	16	3.184	0.0744
Poor	41	51.25	16	25		
**Lymphovascular invasion**						
Absence	35	43.75	25	10	12.81	0.0003**
Presence	45	56.25	14	31		
**Perineural invasion**						
Absence	31	38.75	14	17	0.2609	0.6095
Presence	49	61.25	25	24		
**Tumor invasion**						
T1-2	36	45	24	12	8.41	0.0037**
T3-4	44	55	15	29		
**Lymph node metastasis**						
N0-1	40	50	25	15	6.054	0.0139*
N2-3	40	50	14	26		
**TNM stage**						
I/II	38	47.5	14	24	4.108	0.0427*
III	42	52.5	25	17		
**CEA, ng/ml**						
< 5	44	55	24	20	1.314	0.2516
≥ 5	36	45	15	21		
**Preoperative CTCs** status						
Negative	34	42.5	23	11	8.451	0.0036**
Positive	46	57.5	16	30		

Abbreviations: CEA, carcinoembryonic antigen; CTCs, circulating tumour cell; TNM, tumour-node-metastasis. *P < 0.05, **P < 0.01

### Exosome isolation, characterization, and treatment

Exosomes were isolated and purified from CRC cell culture-conditioned medium (CM) or CRC patient serum using ultracentrifugation, following a previously established exosome extraction method [4]. The quantification of exosomal protein was conducted using the BCA Protein Assay Kit (Beyotime Biotechnology, China). For transmission electron microscopy analysis, exosomes were fixed with 2% paraformaldehyde and observed under a transmission electron microscope (H-7500, Hitachi, Japan) [4]. Tracking of the quantity and size of exosomes was performed using the Nanosight NS 300 system (NanoSight Technology, Malvern, UK); the resulting data were analyzed using the NTA analytical software (version 2.3).

### Animal models

Six-week-old male athymic BALB/c-nu/nu mice were purchased from Beijing Vital River Laboratory Animal Technology (Beijing, China) and maintained in a specific pathogen-free environment. All animal study protocols were reviewed and approved by the Institutional Animal Care and Use Committee of Changshu No. 1 People’s Hospital Affiliated with Soochow University.

For the orthotropic metastasis assay, nude mice were anesthetized before laparotomy was performed to expose the ceca. Subsequently, 2 × 10^6^ CRC cells were injected into the mesentery at the tail end of the cecum. To investigate the role of exosomes in tumor metastasis, 10 µg of CRC-derived exosomes were intravenously injected every three days following the CRC cell implantation.

For ADAM17 inhibitor treatment, the ADAM17 selective inhibitor JG26 was administered intravenously (IV) at a dose of 3 mg/kg, while the ADAM17 oral inhibitor aderbasib was administered orally (PO) at a dose of 30 mg/kg every three days following CRC cell implantation. After 60 days, mice were euthanized, and liver and lung metastases were quantified. The Evans blue index, calculated as the amount of dye in the lung/liver relative to the weight of the lung/liver tissue, was determined as previously described [17]. To detect circulating tumor cells (CTCs) in the blood of mice, a cardiac puncture was performed to collect 1 mL blood samples; these samples were then centrifuged and mixed with EDTA. Subsequently, the cells and plasma were separated. The cells were then resuspended in PBS for CTC detection [7]. To assess metastasis, sections from the liver and lung tissues were stained with hematoxylin and eosin (H&E), and 10 pathological sections from different parts of the liver and lungs of each animal were randomly selected to determine the number and maximum diameter of metastatic nodules in the lungs and livers.

### Cell culture and reagents

Human colorectal carcinoma HCT-116 cells were purchased from the Cell Bank of the Shanghai Institute of Biochemistry and Cell Biology (Shanghai, China). The cells were cultured in Dulbecco’s Modified Eagle Medium supplemented with 10% fetal bovine serum (FBS), 100 U/mL penicillin, and 100 µg/mL streptomycin (Invitrogen, Grand Island, NY, USA). Human umbilical vein endothelial cells (HUVECs), human pulmonary microvascular endothelial cells (HPMECs), and endothelial cell medium (ECM) supplemented with low-serum growth supplement were purchased from ScienCell (USA) [17]. The FBS used for CM exosome collection, exosome isolation, and endothelial cell treatment was separated from exosomes via overnight centrifugation at 100,000 ×*g* and 4 °C. All cell lines were cultured in a humidified atmosphere with 5% CO_2_ at 37 °C. Anti-human neutralizing IL-6 antibodies were obtained from MedChem Express (China). Recombinant human IL-6 (R&D Systems) was dissolved in PBS containing 0.1% BSA to a final concentration of 50 ng/mL [7]. Exosomes were pre-incubated with 2 µg/mL annexin V for 2 h before co-culture with HUVECs.

### Endothelial permeability and trans-endothelial invasion assays

To assess the permeability of the treated HUVEC and HPMEC monolayer membranes (average pore size, 0.4 μm; BD Biosciences, Franklin Lakes, NJ, USA), we evaluated the transfer of rhodamine B isothiocyanate-dextran (20 mg/mL, average MW ∼ 70,000; Sigma-Aldrich, USA), in 40 µL medium aliquots over time.

For the cell invasion assay, we used 24-well Transwell plates (pore size, 8 μm; Corning) pre-coated with Matrigel (Falcon 354,480; BD Biosciences). A total of 1 × 10^5^ cells were suspended in 500 µL RPMI 1640 with 1% FBS and added to the upper chamber, while 750 µL RPMI 1640 containing 10% FBS was added to the bottom chamber. After 48 h, cells that had migrated through and adhered to the lower surface of the membrane were fixed with 4% paraformaldehyde and stained with 0.5% crystal violet. Cell counting and photography were conducted in five optical microscopy fields (×100 magnification), with all experiments repeated thrice. Finally, the transendothelial invasion test was employed to detect green fluorescent protein (GFP)-expressing CRC cells that invaded the HUVEC and HPMEC monolayers with or without exosome treatment.

### RTCA system and transepithelial electrical resistance analysis

The cell index (CI) of the HUVECs was determined using the iCELLigence system (Roche Applied Science, Germany; ACEA Biosciences, Indianapolis, IA, USA), following previously described methods for RTCA CI computation [17]. The CI serves as a quantitative measure of the cell status in each well, including the degree of cell adhesion. The normalized cell index at a specific time point was calculated by dividing the CI value by the value at the reference time point.

Transepithelial electrical resistance (TEER) was measured using an electric resistance system ERS-2 (Millipore), according to the manufacturer’s instructions. Briefly, HUVECs and HPMECs were cultured on Millicell filters (0.33 cm^2^ area; 0.4 μm pore diameter; 6.5 mm diameter), with the culture medium being replaced before TEER measurement. To calculate the actual resistance of the cell monolayer, the mean resistance of the filters without cells was subtracted from the monolayer measurement, thereby correcting for the difference between the filter and the monolayer areas. The values measured immediately after treatment were set at 100% to enable normalization of the results.

### Flow cytometry

For flow cytometry analysis, monolayers of exosome-treated HUVECs and HPMECs were detached from culture flasks by incubation on ice before being washed twice in PBS. Detached cells were resuspended in 200 µL of PBS containing 1% BSA (Sigma-Aldrich) and incubated for 30 min at 4 °C with the following antibodies: PE-conjugated anti-CD144, PE Mouse IgG1, and κ Isotype Control (BD Biosciences). After washing, the cells were analyzed using a FACScan laser flow cytometer, BD FACS AriaII (BD Biosciences). Mean channel fluorescence was expressed as FACS arbitrary values. Finally, the cells were gated using forward vs. side scatter to exclude dead cells and debris.

### Immunofluorescence and immunohistochemistry staining

For immunofluorescence and immunohistochemistry staining, tissue Sect. (4 μm thick) or cells grown on coverslips were fixed with formalin (containing 0.1% diethylpyrocarbonate) for 20 min. Subsequently, the samples were washed with PBS. After permeabilization with 0.4% pepsin for 5–120 s, a blocking buffer (3% BSA) was added. Samples were incubated with primary antibodies, followed by staining with secondary antibodies. Sections and cells were observed, and images were captured using an Olympus BX53 fluorescence microscope. Finally, the expression levels of VE-cadherin, p120, E-cadherin, and vimentin were quantified.

For the cell membrane protein internalization assay, antibodies against VE-cadherin, ZO-1, and occludin were dialyzed into ECM containing 20 mM HEPES and 3% BSA. Subsequently, the dialyzed antibodies were incubated with HUVEC cultures at 4 °C for 1 h. Unbound antibodies were then removed by rinsing cells in ice-cold ECM. Cells were incubated with exosomes in the presence of 100 µM chloroquine. Notably, chloroquine pretreatment prevents endosome–lysosome acidification, thereby allowing cell membrane protein internalization but preventing lysosomal degradation. After treatment, cells were rinsed, fixed, and processed for immunofluorescence labeling.

### Western blot and quantitative proteomic analyses

To prepare cell membrane proteins, vascular endothelial cells were collected via centrifugation at 500 × *g* for 5 min, followed by washing with PBS. The cell pellet was then lysed using the Mem-PER Plus Membrane Protein Extraction Kit, according to the manufacturer’s instructions (Thermo Fisher Scientific). The extracts were stored at -80℃ until use.

For western blot analysis, total protein was extracted from the exosomes and HUVECs using RIPA lysis buffer and assessed as previously described [18]. For quantitative protein analysis, exosomes were labeled with iTRAQ reagents using an iTRAQ multiplex kit (AB Sciex, USA) and analyzed as previously described [19]. Labeled samples were separated and automatically spotted onto a MALDI plate; mass spectra were then acquired using an AB Sciex TOF/TOF 5800 system. All tandem mass spectrometry data were analyzed using MASCOT and Protein Pilot software (version 4.5; AB Sciex) to identify and quantify the corresponding proteins in different groups (Table S2). Protein identification was considered correct based on the selection criteria [19].

### DNA constructs and RNA interference

To inhibit ADAM17 expression in CRC cell-derived exosomes, ADAM17-directed siRNAs were employed. Human ADAM17-specific siRNA (5′-TGAGGCAGTCTCTCCTATTCCTGACCAGC-3′) and nonsense siRNA (5′-TGACCACCCTGACCTACGGCGTGCAGTGC-3′) were obtained from RiboBio (Guangzhou, China). Cells were transfected with ADAM17-siRNA, and nonsense (scrambled) control siRNA using a liposome-based method for 48 h; then, the exosomes were collected for subsequent analyses.

### Statistical analysis

Experimental data were analyzed using the student’s *t*-test and one-way analysis of variance (ANOVA) to identify significant differences between two groups. A Chi-square test was applied to compare the relationship between CirExo-ADAM17 and clinicopathological features (such as sex, age, TNM stage, and metastasis) in patients with CRC who underwent surgical excision. A Kaplan–Meier survival curve was then used to evaluate the relationship between CirExo-ADAM17 expression and survival outcomes in patients with CRC. Log-rank analysis was employed to test for significance, and univariate survival analysis was performed to evaluate the effect of CirExo-ADAM17 on CRC prognosis. Correlations between normally distributed data were analyzed using Pearson or Spearman analysis. Data are presented as the mean ± standard deviation. Western blot analysis was repeated three times, and the results were quantified using ImageJ software. Statistical analyses were performed using the SPSS software (version 13.0; SPSS Inc., Chicago, IL, USA). A *P*-value < 0.05 was considered statistically significant. The remaining of the methods are provided in the supplementary materials.

## Results

### Exosomal ADAM17 serum levels negatively correlate with vascular VE-cadherin expression at the tumor-invasive front

**Fig. 1:**
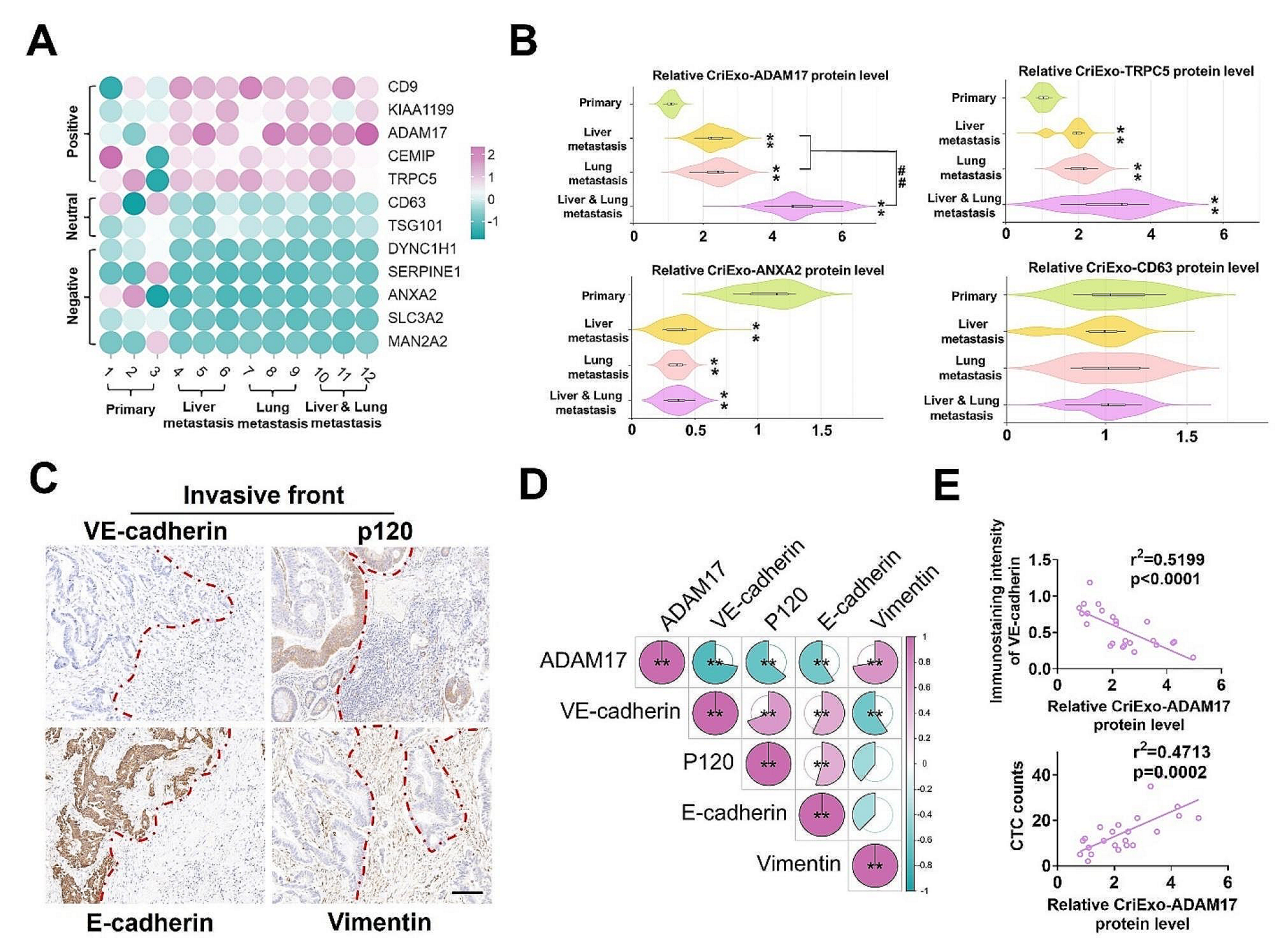
High levels of exosomal ADAM17 in the serum correlate with lower VE-cadherin levels at the tumor invasive front in patients with metastatic colorectal cancer (CRC). (**a**) Proteomic analysis of serum-derived exosomal proteins differentially expressed between non-metastatic (primary), liver metastatic, lung metastatic, and both liver and lung metastatic patients with CRC. The resultant heatmap shows the differentially expressed exosomal proteins based on quantitative mass spectrometry values; all protein analysis was performed in technical triplicate (* false discovery rate < 0.05 determined via ANOVA). Hierarchical clustering was performed on protein expression levels (*n* = 3) using the Spearman’s rank correlation coefficient to identify similarities. (**b**) Relative CirExo-ADAM17, TRPC5, ANXA2, and CD63 protein levels in non-metastatic (primary), liver metastatic, lung metastatic, and both liver and lung metastatic patients with CRC (*n* = 6). (**c**) Representative immunohistochemistry staining for VE-cadherin, p120, and EMT (E-cadherin and vimentin) in the invasive front of tumors from patients with CRC (scale bar = 100 μm). (**d**) Correlation analysis of cirExo-ADAM17 protein levels and immunostaining intensity of VE-cadherin, p120, E-cadherin, and vimentin at the invasive front. (e) Linear regression between the expression of CirExo-ADAM17 protein, immunostaining intensity of VE-cadherin at the invasive front, and total CTC count, respectively. Data are expressed as mean ± standard deviation. **P < 0.01, compared with the primary group; ##*P* < 0.01, compared with the liver metastasis and lung metastasis groups

The specific proteins packaged within tumor-derived exosomes play a critical role in the occurrence of metastasis at target organs [20]. In the current study, we expanded our clinical sample from our preliminary study and employed quantitative mass spectrometry to compare exosomal proteins. Ultimately, this revealed upregulation of ADAM17 in the serum of patients with multiple metastases of CRC (liver/lung metastasis) compared with that of other serum exosomal proteins associated solely with liver and lung metastasis in patients with non-metastasized CRC and those with other CRC metastases (Fig. 1a, b). Notably, ADAM17 emerged as a prominent exosomal protein in liver metastasis-derived exosomes, exhibiting low expression levels in exosomes from patients without metastasis and healthy donors (Table 1; Table S1). Kaplan–Meier univariate survival analysis indicated a relationship between CirExo-ADAM17 expression and poor postoperative prognosis (*P* < 0.05; Fig. S1).

The process of breaching the vascular barrier is important in cancer metastasis [5]. Notably, ADAM17 regulates the vascular endothelial cell barrier and promotes tumor growth [21], while VE-cadherin serves as a crucial adhesion protein that inhibits tumor hematogenous metastasis [5]. We explored the VE-Cadherin/p120 expression and EMT markers in sections specimens from patients with CRC and observed a low expression of E-Cad and a high expression of vimentin near the invasive front. Furthermore, a high vimentin expression was associated with a low expression of VE-Cadherin and p120 near the invasive front, indicating higher permeability (Fig. 1c and S2a). Based on the exosomal ADAM17 levels in the serum of patients with tumors, exosomal ADAM17 expression was negatively correlated with VE-cadherin, p120, and E-cadherin levels at the tumor invasive front and positively correlated with the expression of tumor blood metastatic protein vimentin (Fig. 1d-e and S2B). Additionally, the exosomal ADAM17 expression was positively correlated with the CTC count in patients with CRC (Fig. 1e). Overall, these findings suggest that CRC-derived exosomal ADAM17 promotes hematogenous tumor metastasis by regulating blood vessel permeability.

### EMT-CRC exosomes increase vascular endothelial cell permeability and facilitate invasion in a VE-cadherin-dependent manner

To explore the effect of EMT-CRC-derived exosomes on the vascular barrier, we employed a CRC cell line (HCT116) with epithelial properties and induced EMT using IL-6, a method established in prior studies [6, 7]. Following IL-6 treatment, the EMT characteristics of the HCT116 cells were confirmed (Fig. S3a-d). After acquiring a highly invasive phenotype, traversing the blood vessel barrier becomes a crucial step in hematogenous metastasis. Therefore, we explored the role of exosomes in the intercellular communication between mesenchymal CRC cells and blood vessel endothelial cells.

**Fig. 2:**
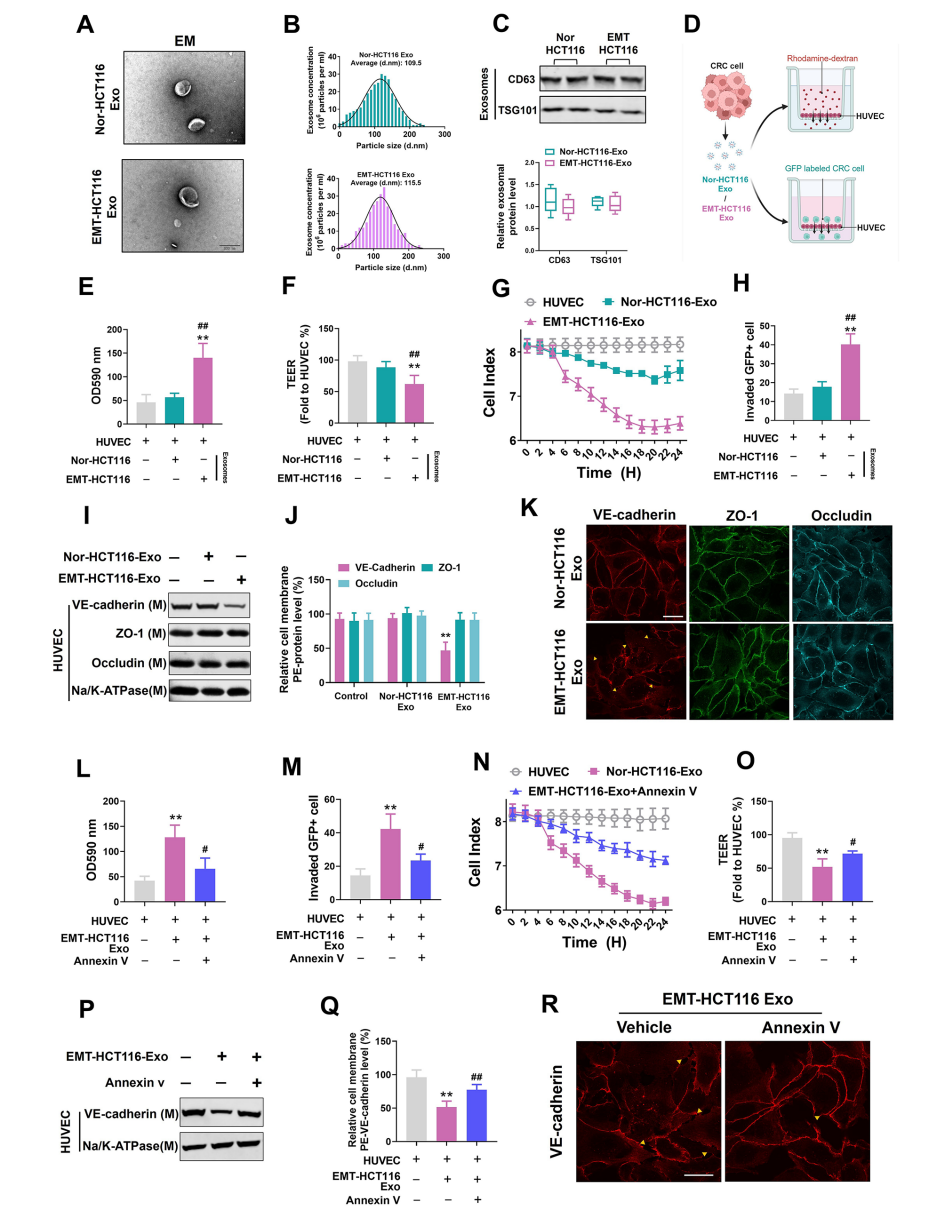
EMT-CRC exosomes increase vascular endothelial cell permeability and facilitate invasion by disrupting VE-cadherin expression. (**a**) Electron microscope images of exosomes isolated from the conditioned medium of cultured Nor-HCT116 and EMT-HCT116 cells (scale bar = 200 nm). (**b**) Nanoparticle tracking analysis of isolated exosomes. (**c**) Western blot analysis of exosomal membrane markers CD63 and TSG101. (**d**) Schematic illustration of the permeability analysis of human umbilical vein endothelial cells (HUVECs). Upper panel: Permeability assessment was conducted using rhodamine-labeled dextrose molecular probes capable of passing through a developing HUVEC monolayer membrane on a filter. Lower panel: The number of tumor cells that infiltrated the HUVEC monolayer on the filter was calculated. (**e**) HUVEC monolayer membranes were pretreated with Nor-HCT116/EMT-HCT116-derived exosomes (50 µg; 10 µg/mL per 1 × 10^5^ cultured cells) for 24 h. Then, rhodamine–dextran was added to the upper chamber and incubated for 60 min. Subsequently, dextran content was detected in the lower chamber at an optical density (OD) of 590 nm. (**f, g**) HUVEC monolayer membranes were pretreated with Nor-HCT116/EMT-HCT116-derived exosomes (50 µg; 10 µg/mL per 1 × 10^5^ cultured cells) for 24 h; then, the TEER and CI values were calculated. (**h**) HUVECs were incubated with Nor-HCT116/EMT-HCT116-derived exosomes (50 µg; 10 µg/mL per 1 × 10^5^ cultured cells) for 24 h. The number of tumor cells that invaded through the HUVEC monolayer was determined. (**i, j, k**) HUVECs were incubated with Nor-HCT116/EMT-HCT116-derived exosomes (50 µg; 10 µg/mL per 1 × 10^5^ cultured cells) for 4 h. The cell membrane VE-cadherin, ZO-1, and occludin levels were measured using western blotting, flow cytometry, and immunofluorescence (scale bar = 25 μm). Data are expressed as mean ± standard deviation (*n* = 5). **P < 0.01, compared with the control group; ##*P* < 0.01, compared with the Nor-HCT116 group. (**l, m**) HUVECs were incubated with exosomes derived from EMT HCT116 or EMT HCT116 + Annexin V cells. Dextran content was detected (OD 590 nm) in the lower chamber, and the number of tumor cells invading the HUVEC monolayer was determined. (**n, o**) HUVECs incubated with exosomes derived from EMT HCT116 or EMT HCT116 + Annexin V cells. TEER and CI values were calculated. (**p–r**) HUVECs were incubated with exosomes derived from EMT HCT116 or EMT HCT116 + Annexin V cells. Cell membrane VE-cadherin levels were measured using western blotting, flow cytometry, and immunofluorescence (scale bar = 25 μm). Data are expressed as mean ± standard deviation (*n* = 5). **P < 0.01, compared with the control group; #*P* < 0.05, ##*P* < 0.01, compared with the EMT-HCT116 group

Exosomes isolated from the culture media of control and EMT-HCT116 cells displayed a typical disc-shaped morphology, with an approximate diameter of 100 nm (Fig. 2a). NanoSight analysis of the isolated exosomes revealed median diameters of 109.5 and 115.5 nm, respectively (Nor-HCT116 and EMT-HCT116) (Fig. 2b). Exosomal membrane markers, TSG101 and CD63, were detected using western blotting to confirm the effective isolation of exosomes (Fig. 2c).

Subsequently, HUVECs were treated with exosomes purified from normal/EMT HCT116 cells. In vitro, permeability assays were conducted to determine if exosomes secreted by EMT-CRC cells regulate vascular permeability. Exosomes from EMT-HCT116 cells enhanced vascular permeability compared to the exosomes from control cells (Fig. 2d, e). Moreover, resistance and cell index analysis revealed that exosomes derived from EMT CRC cells significantly reduced the integrity of the vascular endothelial barrier (Fig. 2f, g). The transendothelial cell invasion assay confirmed these findings by detecting the presence of GFP-labeled tumor cells penetrating the HUVEC monolayer (Fig. 2h).

Western blotting, flow cytometry, and immunofluorescence assays showed a significant decrease in VE-cadherin cell membrane localization level in HUVECs treated with EMT-HCT116 exosomes, whereas ZO-1 and occludin levels remained unaffected (Fig. 2i-k). Nonetheless, when HUVECs were treated with an exosome internalization inhibitor, Annexin V [7], EMT-HCT116-derived exosomes failed to induce increased permeability (Fig. 2l-o). Additionally, western blotting, flow cytometry, and immunofluorescence analysis indicated a restoration of VE-cadherin/p120 expression in Annexin V-treated HUVECs (Fig. 2p-r). Overall, our results suggest that EMT-HCT116-secreted exosomes can alter vascular endothelial permeability.

### Exosomal ADAM17 disrupts the vascular endothelial cell barrier by targeting VE-cadherin

**Fig. 3:**
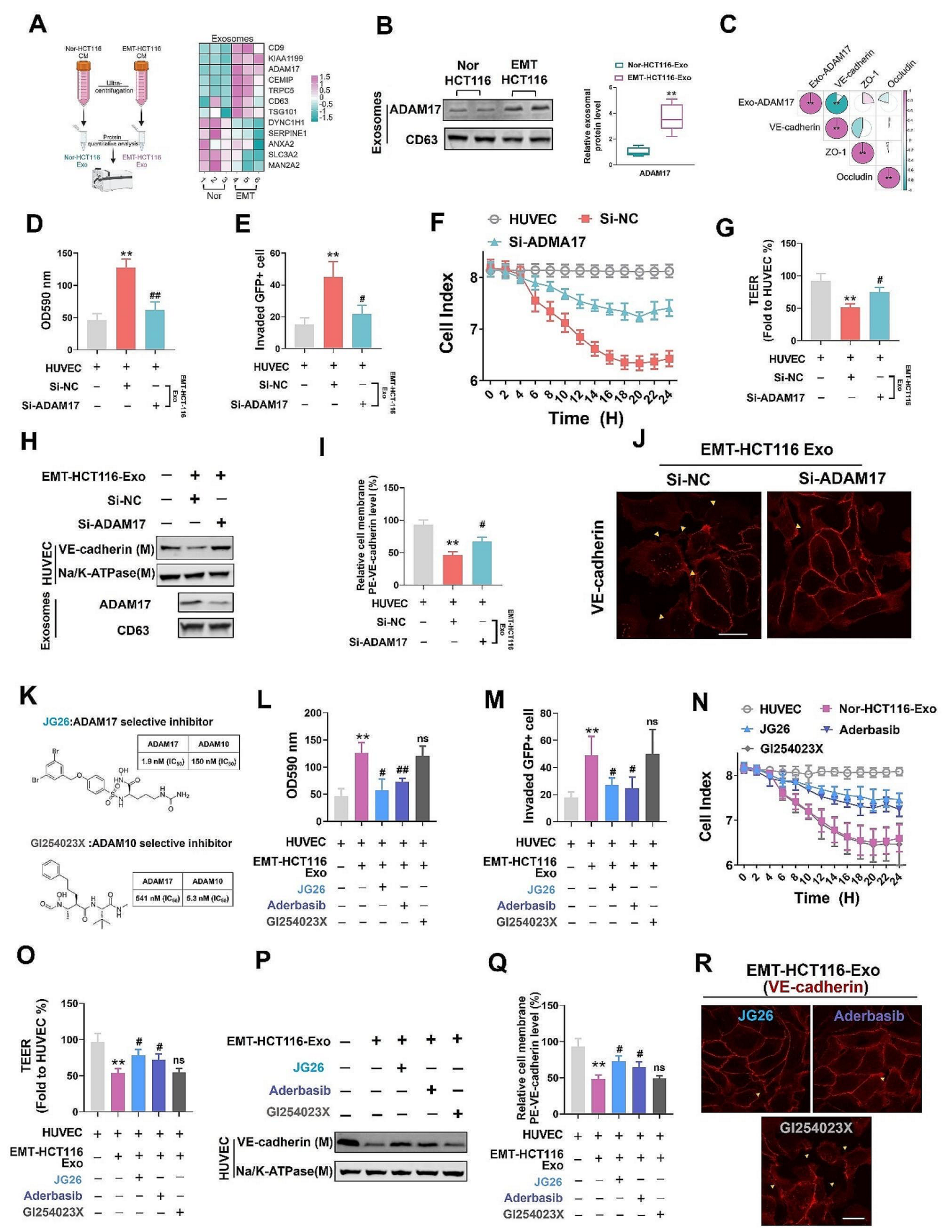
Exosomal ADAM17 induces vascular endothelial barrier damage by targeting VE-cadherin. (**a**) Heatmap showing the expression change of proteins in exosomes secreted by Nor-HCT116 and EMT-HCT116 cells based on human serum exosome data. (**b**) Western blot analysis of ADAM17 and the exosomal marker CD63. (**c**) Correlation analysis of Exo-ADAM17 protein levels and the protein levels of VE-cadherin, zo-1 and occludin on treated human umbilical vein endothelial cell (HUVEC) membranes. (**d, e**) HUVECs were incubated with exosomes derived from EMT HCT116 cells (Si-NC and Si-ADAM17 transfected). Dextran was detected (OD 590 nm) in the lower chamber and the number of tumor cells invading the HUVEC monolayer was determined. (**f, g**) HUVECs were incubated with exosomes derived from EMT HCT116 cells (Si-NC and Si-ADAM17 transfected); TEER and CI values were calculated. (**h–j**) HUVECs were incubated with exosomes derived from EMT HCT116 cells (Si-NC and Si-ADAM17 transfected); cell membrane VE-cadherin levels were measured using western blotting, flow cytometry, and immunofluorescence (scale bar = 25 μm). Data are expressed as the mean ± standard deviation (*n* = 5). **P < 0.01, compared with the control group; #*P* < 0.05, ##*P* < 0.01, compared with the Si-NC group. (**k**) Exosomes were pre-incubated with ADAM17 selective inhibitor-JG26 (25 nM), ADAM17 oral inhibitor aderbasib (1 µM), and ADAM10 selective inhibitor-GI254023 × (25 nM) for 2 h before culturing with HUVECs. (**l, m**) Dextran content was detected (OD 590 nm) in the lower chamber, and the number of tumor cells invading the HUVEC monolayer was determined. (**n, o**) TEER and CI values. (**p–r**) Cell membrane VE-cadherin levels were determined using western blotting, flow cytometry, and immunofluorescence (scale bar = 25 μm). Data are expressed as mean ± standard deviation (*n* = 5). **P < 0.01, compared with the control group; #*P* < 0.05, ##*P* < 0.01, compared with the EMT-HCT116-Exo group

Based on the differentially expressed proteins identified through preliminary clinical screening, quantitative mass spectroscopy was employed to analyze the proteins in Nor-HCT116 and EMT-HCT116 exosomes. The corresponding results revealed that ADAM17 was highly expressed in EMT-HCT116 exosomes (Fig. 3a, b), which was consistent with our previous findings in different CRC cell lines. In particular, exosomal ADAM17 is highly expressed in metastatic cell lines, such as SW620 and Lovo, and is closely related to tumor metastasis [14]. In the present study, we treated HUVECs with different amounts of exosomal ADAM17 and found that exosomal ADAM17 was closely related to changes in VE-cadherin on the cell membrane of vascular endothelial cells (Fig. 3c). Further siRNA interference experiments demonstrated that suppressing ADAM17 expression significantly reduced the permeability of HUVECs (Fig. 3d, e) and mitigated endothelial barrier dysfunction (Fig. 3f, g), both of which were induced by EMT-HCT116 exosomes. Notably, ADAM17 interference significantly reduced VE-Cadherin levels in HUVEC membranes, otherwise induced by EMT-HCT116 exosomes (Fig. 3h-j and S4a). Similarly, si-ADAM17 positive and negative validation in HPMECs demonstrated that EMT-HCT116-derived exosomal ADAM17 promotes HPMEC permeability and tumor cell adhesion by targeting the VE-cadherin-mediated vascular endothelial barrier (Fig. S5a-h).

Next, we conducted comparative experiments using a selective inhibitor of ADAM17, JG26 [22], an oral inhibitor of ADAM17, aderbasib [23], and a selective inhibitor of ADAM10, GI254023X [24] (Fig. 3k). Compared with the selective inhibitor of ADAM10 (GI254023X), the ADAM17 inhibitors JG26 and aderbasib effectively reduced the permeability and tumor cell adhesion induced by EMT-HCT116 exosomes (Fig. 3l, m). Simultaneously, treatment with these ADAM17 inhibitors reduced the enhanced VE-cadherin expression induced by EMT-HCT116 exosomes in endothelial cells and the chemical and endothelial barrier permeability of HUVECs (Fig. 3n-r and S4b) and HPMECs (Fig. S5i-l). In summary, these findings indicate that EMT-HCT116-secreted exosomal ADAM17 promotes vascular endothelial permeability by targeting VE-cadherin.

### Exosomal ADAM17 promotes CRC metastasis by increasing vascular permeability in vivo

**Fig. 4:**
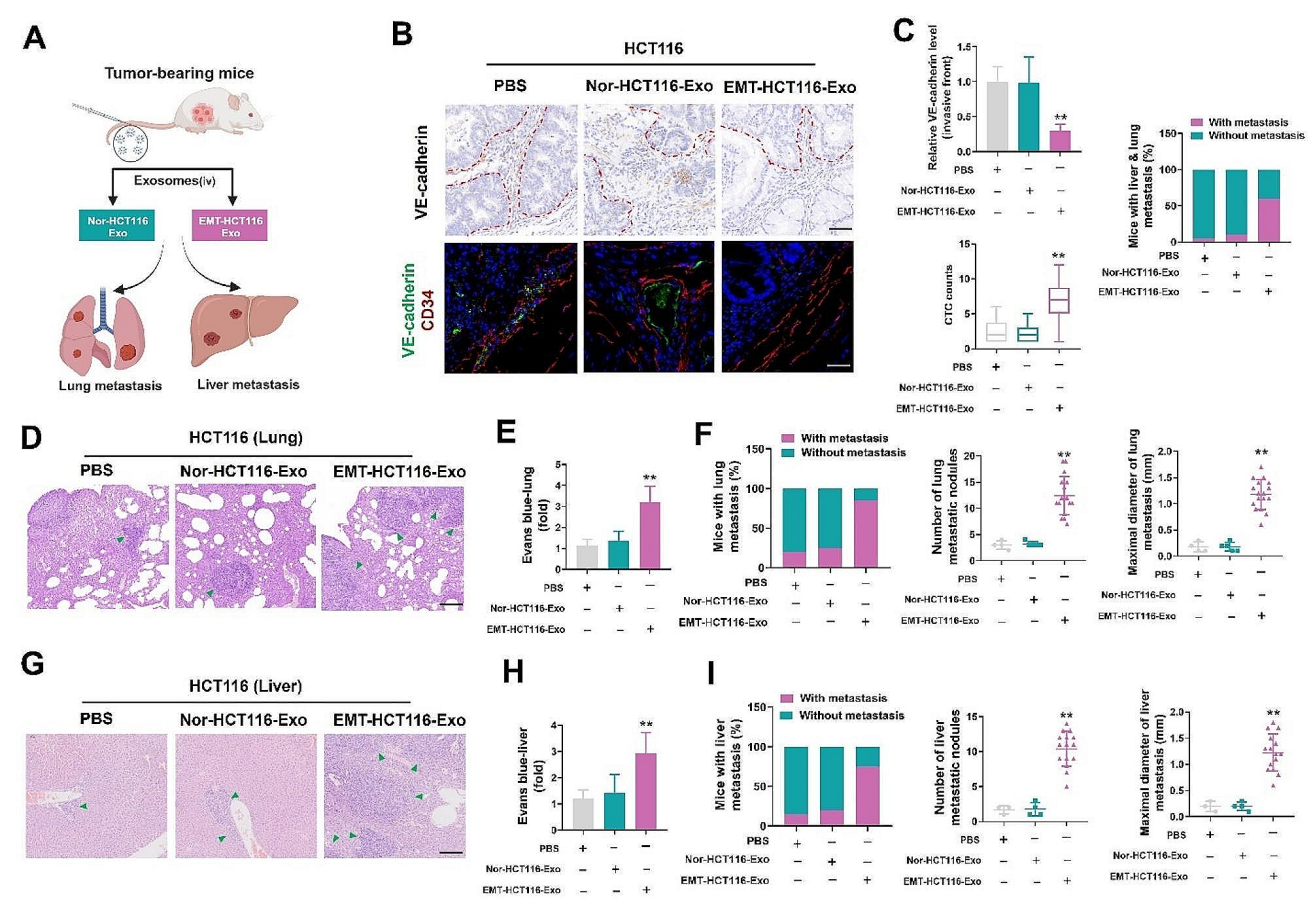
EMT-CRC exosomes enhance tumor metastasis by regulating VE-cadherin expression in vivo. (**a**) Schematic illustration of the tumor metastasis experiments used to assess Nor-HCT116-Exo- and EMT-HCT116-Exo-treated mice. (**b**) Representative immunohistochemistry staining of VE-cadherin and immunofluorescence staining of CD34/VE-cadherin at the invasive front in mice with CRC (black scale bar = 50 μm, white scale bar = 25 μm). (**c**) Analysis of Nor-HCT116-Exo and EMT- HCT116-Exo treated mice, showing relative VE-cadherin levels at the invasive front (*n* = 10), CTC counts (*n* = 20), and percentage of mice with both lung and liver metastasis. (**d**) Representative H&E staining results of lung sections from Nor-HCT116-Exo- and EMT-HCT116-Exo-treated mice. Arrows indicate tumor nodules. Scale bar = 250 μm. (**e**) Evans blue accumulation in the lung parenchyma (*n* = 8). (**f**) The percentage of mice with lung metastasis, alongside the number and maximal diameter of metastatic nodules in the lungs of Nor-HCT116-Exo- and EMT- HCT116-Exo-treated mice. (**g**) Representative H&E staining results of liver sections from Nor-HCT116-Exo- and EMT-HCT116-Exo-treated mice. Arrows indicate tumor nodules. Scale bar = 250 μm. (**h**) Evans blue accumulation in the liver parenchyma (*n* = 8). (**I**) Percentage of mice with liver metastasis, alongside the number and maximal diameter of metastatic nodules in the liver of Nor-HCT116-Exo- and EMT-HCT116-Exo-treated mice. Data are expressed as mean ± standard deviation. *P < 0.05, **P < 0.01, compared with the control group

To investigate the role of exosomal ADAM17 in the disruption of blood vessel barriers and induction of cancer metastasis in vivo, control cell lines (HCT116) were implanted into murine ceca, and Nor-HCT116 and EMT-HCT116 exosomes were administered to assess the effects of exosomal proteins on vascular barrier function and tumor metastasis, respectively (Fig. 4a).

Compared with that in the Nor-HCT116 exosome treatment group, EMT-HCT116 exosomes significantly reduced VE-cadherin expression at the invasive front of CRC tumors (Fig. 4b, c); this was accompanied by an increase in CTCs in mouse blood and an increase in the proportion of mice with both lung and liver metastasis (Fig. 4c).

To confirm the in vivo effect of vascular permeability, Evans blue albumin was injected into the tail veins of orthotopic-bearing mice. Notably, more Evans blue was extravasated into the lungs and liver in mice treated with EMT-HCT116 exosomes than in those treated with Nor-HCT116 exosomes (Fig. 4e, h), indicating significantly higher vascular permeability at the primary tumor site. Subsequently, the mice treated with the EMT-HCT116 exosome displayed higher rates of lung and liver metastases, along with an increased number and size of metastatic nodules, compared to mice treated with the Nor-HCT116 exosomes (Fig. 4d, f, g, i).

**Fig. 5:**
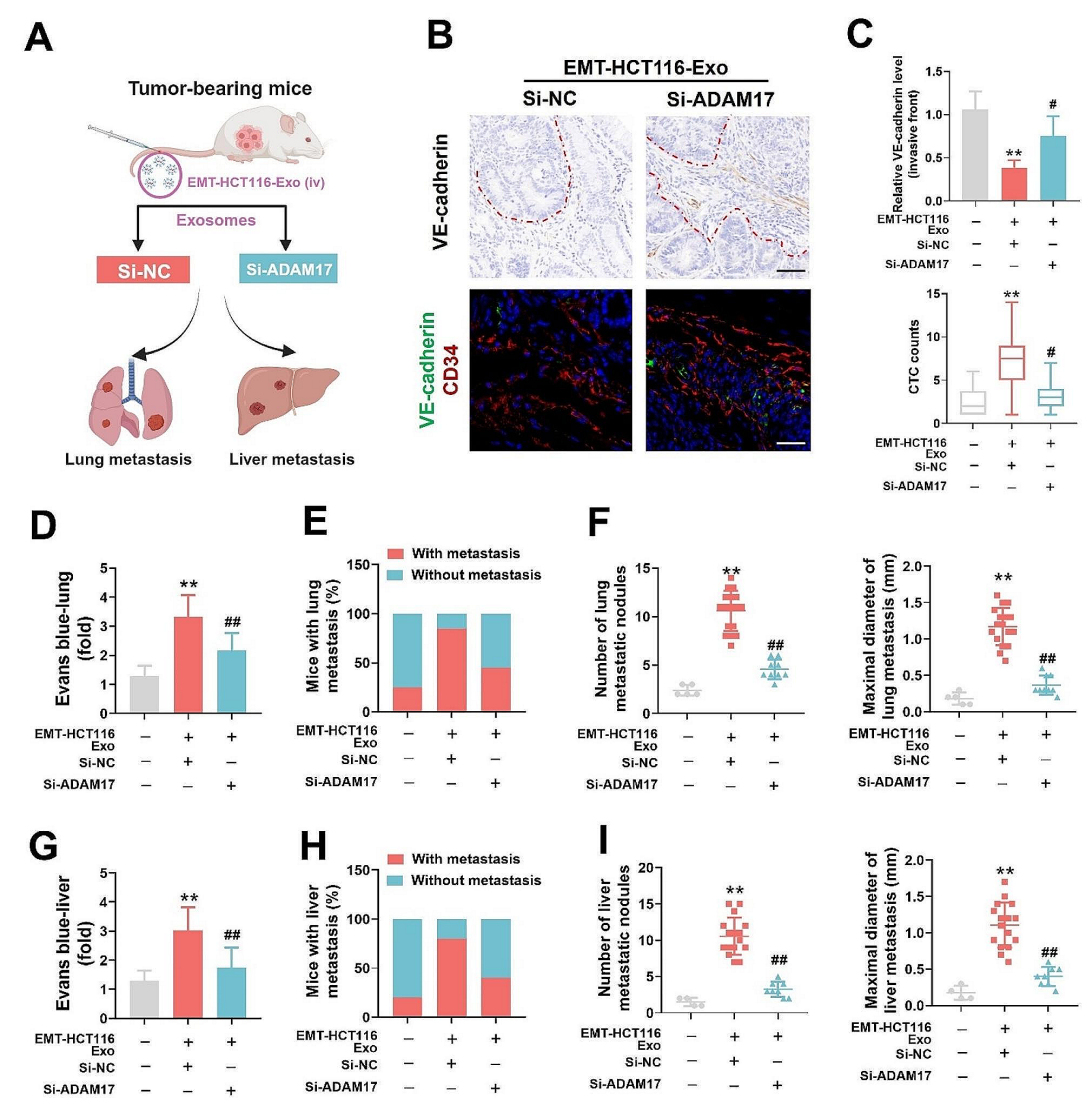
Knocking down exosomal ADAM17 effectively reduces colorectal cancer (CRC) metastasis in vivo. (**a**) Schematic showing the tumor metastasis experiments used to assess EMT-HCT116-Exo-treated mice (Si-NC and Si-ADAM17 transfected). (**b**) Representative immunohistochemistry staining for VE-cadherin and immunofluorescence staining of CD34/VE-cadherin at the invasive front in mice with CRC (black scale bar = 50 μm, white scale bar = 25 μm). (**c**) Relative VE-cadherin level at the invasive front (*n* = 10) and CTC counts (*n* = 20) in EMT-HCT116-Exo-treated mice (Si-NC and Si-ADAM17 transfected). (**d**) Representative H&E staining results of lung sections from EMT-HCT116-Exo-treated mice (Si-NC and Si-ADAM17 transfected). Arrows indicate tumor nodules. Scale bar = 250 μm. (**e**) Evans blue accumulation in the lung parenchyma (*n* = 8). (**f**) Percentage of mice with lung metastasis, alongside the number and maximal diameter of metastatic nodules in the lungs of EMT-HCT116-Exo-treated mice (Si-NC and Si-ADAM17 transfected). (**g**) Representative H&E staining results of liver sections from EMT-HCT116-Exo-treated mice (Si-NC and Si-ADAM17 transfected). Arrows indicate tumor nodules. Scale bar = 250 μm. (**h**) Evans blue accumulation in the liver parenchyma (*n* = 8). (**i**) Percentage of mice with liver metastasis, alongside the number and maximal diameter of metastatic nodules in the liver of EMT-HCT116-Exo-treated mice (Si-NC and Si-ADAM17 transfected). Data are expressed as mean ± standard deviation. *P < 0.05, **P < 0.01, compared with the control group; #*P* < 0.05, ##*P* < 0.01, compared with the Si-NC group

Next, siRNA interference was applied to EMT-HCT116 cells and isolated exosomes, to treat tumor-bearing mice (Fig. 5a). Compared with that in the si-NC group, si-ADAM17 significantly reversed the EMT-HCT116 exosome-induced decrease in VE-cadherin expression at the invasive front of CRC tumors (Fig. 5b, c); moreover, si-ADAM17 prevented the increase in the number of CTCs (Fig. 5c) and the increased proportion of mice with both lung and liver metastasis, otherwise induced by EMT-HCT116 exosomes (Fig. S6a). Furthermore, si-ADAM17 treatment led to a reduction in the EMT-HCT116 exosome-induced increase in vascular permeability (Fig. 5d, g), lung and liver metastasis rates, and the number and size of metastatic nodules in the lungs and liver (Fig. 5e, f, h, i). Overall, these findings indicate that elevated levels of ADAM17 in CRC cellular exosomes can disrupt vascular integrity and promote hematogenous metastasis in CRC.

### ADAM17 inhibitors effectively reduce CRC metastasis in vivo

**Fig. 6:**
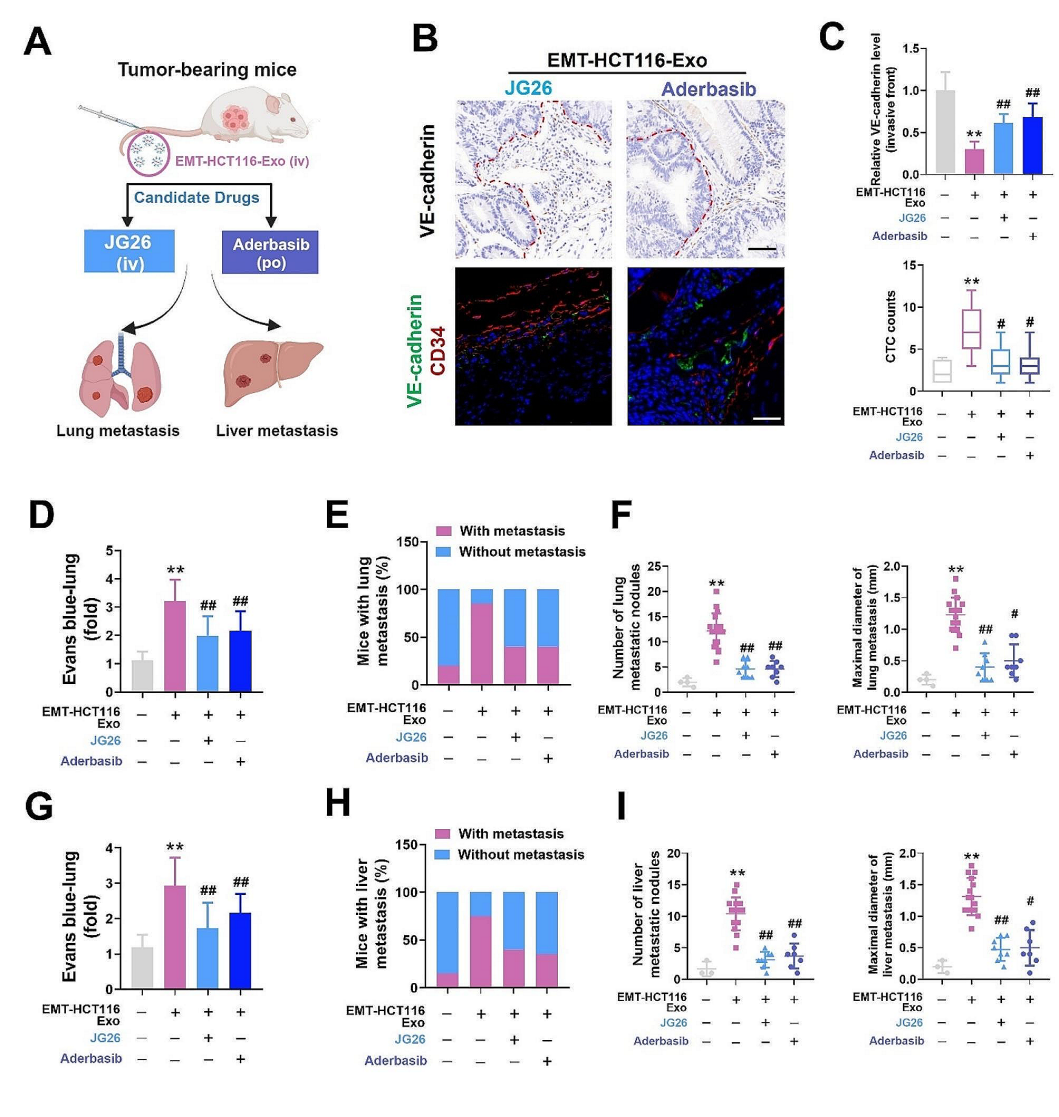
ADAM17 selective inhibitor JG26 and oral inhibitor aderbasib effectively reduce colorectal cancer (CRC) metastasis in vivo. (**a**) Schematic illustration of the tumor metastasis experiments used to assess EMT-HCT116-Exo-treated mice that were administered ADAM17 selective inhibitor JG26 (3 mg/kg, IV) and ADAM17 oral inhibitor aderbasib (30 mg/kg, PO). (**b**) Representative immunohistochemistry staining for VE-cadherin and immunofluorescence staining of CD34/VE-cadherin at the invasive front in mice with CRC (black scale bar = 50 μm, white scale bar = 25 μm). (**c**) Relative VE-cadherin level at the invasive front (*n* = 10) and CTC counts (*n* = 20) in EMT-HCT116-Exo-treated mice (3 mg/kg JG26, IV; 30 mg/kg aderbasib, PO). (**d**) Representative H&E staining results of lung sections from EMT-HCT116-Exo-treated mice (3 mg/kg JG26, IV; 30 mg/kg aderbasib, PO). Arrows indicate tumor nodules. Scale bar = 250 μm. (**e**) Evans blue accumulation in the lung parenchyma (*n* = 8). (**f**) Percentage of mice with lung metastasis, alongside the number and maximal diameter of metastatic nodules in the lungs of EMT-HCT116-Exo-treated mice (3 mg/kg JG26, IV; 30 mg/kg aderbasib, PO). (**g**) Representative H&E staining results of liver sections from EMT-HCT116-Exo-treated mice (3 mg/kg JG26, IV; 30 mg/kg aderbasib, PO). Arrows indicate tumor nodules. Scale bar = 250 μm. (**h**) Evans blue accumulation in the liver parenchyma (*n* = 8). (**i**) Percentage of mice with liver metastasis, alongside the number and maximal diameter of metastatic nodules in the liver of EMT-HCT116-Exo-treated mice (3 mg/kg JG26, IV; 30 mg/kg aderbasib, PO). Data are expressed as mean ± standard deviation. *P < 0.05, **P < 0.01, compared with the control group; #*P* < 0.05, ##*P* < 0.01, compared with the EMT-HCT116-Exo group

To further investigate the potential clinical application of ADAM17 inhibitors to reverse CRC hepatic metastasis, we employed the ADAM17 selective inhibitor JG26 and the oral ADAM17 inhibitor aderbasib, investigating their effects on CRC lung/hepatic metastasis in vivo (Fig. 6a). In vivo drug trials demonstrated that these ADAM17 inhibitors significantly reduced the EMT-HCT116 exosome-induced decrease in VE-cadherin at the invasive front of CRC tumors (Fig. 6b, c); moreover, treatment with these inhibitors reduced the number of CTCs in mouse blood (Fig. 6c) and reduced the proportion of mice with both lung and liver metastasis (Fig. S6b). Furthermore, JG26 and aderbasib treatment led to a reduction in the EMT-HCT116 exosome-induced increase in vascular permeability (Fig. 6d, g), lung and liver metastases, and the number and size of metastatic nodules in the lungs and liver (Fig. 6e, f, h, i). In summary, these results suggest that elevated levels of ADAM17 in CRC cellular exosomes can disrupt vascular integrity and promote hematogenous metastasis in CRC. Nonetheless, they also indicate that ADAM17 selective inhibitors can effectively reduce the hematogenous metastasis of CRC in vivo.

## Discussion

Blood vessel endothelium acts as a barrier against CRC hematogenous metastasis by directly blocking tumor cell penetration [8, 25]. Cancer-derived exosomes are considered major drivers of cancer-induced pre-metastatic niche formation at distant sites [4]. Tumor-derived exosomes carrying miRNAs, mRNA, and long noncoding RNA facilitate cancer-induced vascular permeability and pre-metastatic niche formation [4, 6, 26, 27]. However, the specific roles of exosomal proteins, particularly membrane-bound proteins, in regulating vascular barrier integrity to promote hematogenous metastasis of tumor cells remain unclear. The present study revealed that ADAM17 was significantly upregulated in exosomes derived from patients with metastatic CRC, as well as EMT CRC cells; notably, this protein was found to further act on blood vessel endothelial cells. Specifically, exosomal ADAM17 disrupts VE-cadherin-mediated adhesion, thereby increasing vascular permeability and facilitating CRC cell intravasation, CTC generation, and ultimately metastasis. Furthermore, we determined that exosomal ADAM17 levels in the plasma were associated with CTC counts in patients with CRC. Overall, our study elucidated the function and clinical significance of secreted exosomal ADAM17 in CRC metastasis.

Current research on the role of sheddase enzymes on exosomal surfaces remains limited. Nonetheless, our preliminary research indicated that the exosomal surface protein ADAM17 is closely associated with metastasis in patients with CRC [14]. Notably, the present study expanded the sample size and incorporated a detailed classification of multiple metastases in patients with CRC. Notably, the statistical analysis of clinical data further confirmed the predictive role of exosomal ADAM17 in CRC metastasis. Preliminary results indicated that exosomal ADAM17 promotes the initial stage of CRC metastasis by cleaving E-cadherin in tumor cells [14]. Therefore, this study aimed to determine whether exosomal ADAM17 can disrupt homologous proteins of the E-cadherin family [28], such as VE-cadherin in vascular endothelial cells; additionally, we investigated the role of ADAM17 in mediating endothelial barrier function during CRC hematogenous metastasis. Overall, our study revealed that CRC-derived exosomal ADAM17 effectively blocks VE-cadherin-mediated adhesion to vascular endothelial cells, thereby increasing endothelial barrier permeability and enhancing tumor cell metastasis. Notably, exosomal ADAM17-mediated protein cleavage on the membrane of endothelial cells demonstrates a degree of selectivity, with no significant impact on the tight junction proteins ZO-1 and occludin in endothelial cells [5]. This novel mode of action may indicate some supplementary mechanisms for the initiation stage of ADAM17-associated endothelial cell apoptosis and metastasis [21]. AMAM17 is closely related to the functions of the tetraspanin CD82, a protein that plays an important role in the exosome packaging [29, 30]. However, our proteomic data analysis indicated that CD82 expression did not significantly correlate with ADAM17 (data not shown). Further studies with large sample size remain warranted to analyze the potential molecular mechanisms of specific tetraspanins regulating ADAM17 assembly in colon cancer cells. In this study, we conducted experimental analyses on another important metastasis-associated member of the ADAM family, ADAM10 [31]. The corresponding results revealed that using the selective inhibitor of ADAM17 (JG26) and the selective inhibitor of ADAM10 (GI254023X) to treat EMT-CRC exosome-treated cells did not reverse the VE-cadherin internalization and enhanced barrier permeability induced by EMT-CRC exosomes in vascular endothelial cells. These findings were consistent with the results of ADAM10 quantitative mass spectrometry detection of metastatic patient samples and EMT-CRC cell-derived exosome proteins, which did not show significant differences.

Similarly, we validated the role of EMT-CRC exosomes in promoting CRC metastasis in an in vivo CRC metastasis model and employed siRNA to interfere with ADAM17 expression in these exosomes. These in vivo experiments confirmed that suppressing ADAM17 expression increases VE-cadherin expression at the tumor invasion front, thereby mitigating the upregulation of CRC hematogenous metastasis and pre-metastatic niche formation otherwise induced by ADAM17. Subsequently, we conducted in vivo experiments using two ADAM17 inhibitors to suppress the hematogenous metastasis of CRC. One of these ADAM17 inhibitors, JG26, can effectively inhibit the enzymatic activity of ADAM17 at concentrations below 10 nmol while having no significant effects on other ADAM enzymes [22]. Therefore, JG26 represents a promising ADAM17 inhibitor with good selectivity and a low minimum effective concentration for clinical application as an anti-metastatic drug. In this study, we tested the in vivo pharmacological effects of another ADAM17 inhibitor, aderbasib, on reducing CRC hematogenous metastasis. Although aderbasib requires a high concentration for administration, it exhibits good oral pharmacological activity, offering a new avenue for the development of clinical anti-metastatic drugs.

## Conclusions

Through the development of previous clinical trials, including increased sample size, subdivision of patient metastasis types, and the quantitative identification of EMT-CRC-derived exosome proteins, we identified exosomal ADAM17 as an effective diagnostic factor for hematogenous metastasis in CRC. Both in vitro and in vivo experiments indicated that exosomal ADAM17 promoted CRC hematogenous metastasis by regulating VE-cadherin-mediated adhesion in vascular endothelial cells. However, further analysis is required to explore the selectivity and specific sites of action of ADAM17 on VE-cadherin. Our study illustrated the potential application of two ADAM17 inhibitors for the inhibition of CRC hematogenous metastasis. Specifically, JG26 exhibits strong selectivity for ADAM17 and a low minimum effective concentration, whereas aderbasib displays good oral activity. These two drugs are promising effective ADAM17 inhibitors for CRC hematogenous metastasis. Exosomal ADAM17 plays distinct roles in promoting metastasis during the initial and hematogenous stages of CRC metastasis. Nonetheless, further studies are required to elucidate the role of ADAM17 during other stages of CRC metastasis. Our findings demonstrate the crucial role of exosomal ADAM17 in the hematogenous metastasis of CRC and its potential function as a valuable blood-based biomarker and target for intervention strategies against CRC metastasis.

**Fig. 7:**
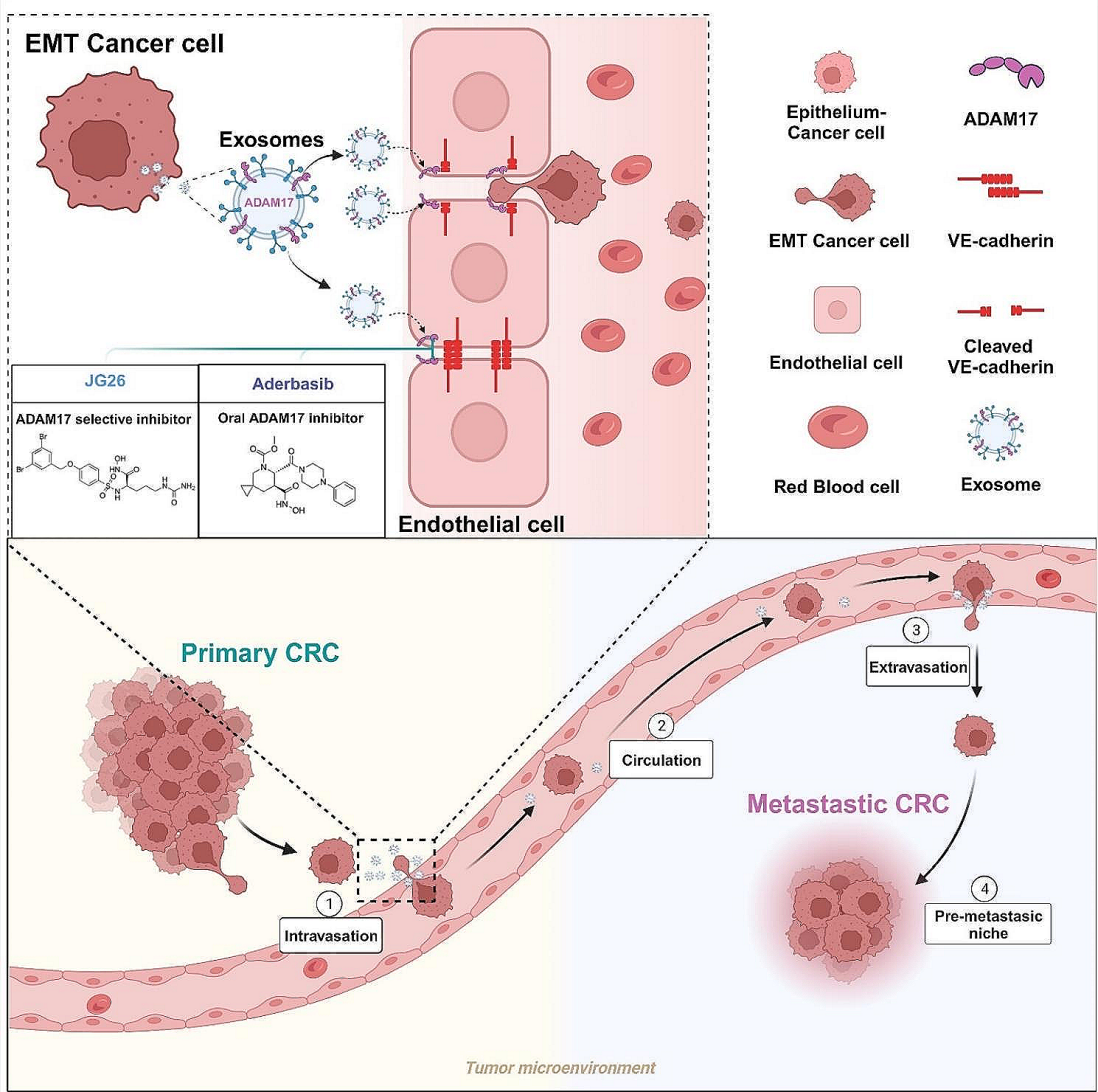
A schematic model showing the functions of exosomal ADAM17 in CRC hematogenous metastasis. The current study indicated the presence of intercellular communication between mesenchymal cancer cells and the vascular endothelium. EMT-CRC-derived exosomal ADAM17 disrupts expression of the adherent junction protein VE-cadherin, thus dysregulating the vascular barrier and facilitating the generation of CTCs and metastasis

### Electronic supplementary material

Below is the link to the electronic supplementary material.

Supplementary Material 1

## Data Availability

All data relevant to the study are included in the article or uploaded as supplementary information. A list of SWISS MODEL accessions can be found in the Supplementary Material.

## Abbreviations

CRC: Colorectal cancer
ADAM17: A disintegrin and metalloproteinase 17
IL-6: Interleukin 6
TNM: Staging Tumor, node, and metastasis staging
CM: Conditioned medium
CTC: Circulating tumor cell
EDTA: Ethylenediaminetetraacetic acid
H&E: Hematoxylin and eosin
HUVEC: Human umbilical vein endothelial cell
HPMEC: Human pulmonary microvascular endothelial cell
ECM: Endothelial cell medium
GFP: Green fluorescent protein
RTCA: Real-time cell analysis
CI: Cell index
TEER: Transepithelial electrical resistance
MCTC: Mesenchymal circulating tumor cell
ANOVA: Analysis of variance
ADAM10: A disintegrin and metalloproteinase domain-containing protein 10
