# Supplementary material for: Tumor-derived exosomal ADAM17 promotes pre-metastatic niche formation by enhancing vascular permeability in colorectal cancer
Source: J Exp Clin Cancer Res. 2024 Feb 27;43:59. doi: 10.1186/s13046-024-02991-3 (PMC10898123; doi:10.1186/s13046-024-02991-3)
Supplement: Supplementary file 1 — Supplementary Material 1 [file 13046_2024_2991_MOESM1_ESM.docx]

**Supplementary Information**

**Tumor-derived exosomal ADAM17 promotes pre-metastatic niche formation by enhancing vascular permeability in colorectal cancer**

Keyu Li^1#^, Wenhua Xue^2#^, Zhihua Lu^4#^, Suo Wang^1^, Jiayao Zheng^1^, Kuangyi Lu^1^, Ming Li^1^, Yang Zong^1^, Feng Xu^1^, Jiamin Dai^1^, Yang Yang^3*^, Jinbing Sun^1*^

**^1^**Department of General Surgery, Changshu No. 1 People's Hospital, Affiliated Changshu Hospital of Soochow University, Changshu, Jiangsu 215500, China

**^2^**Department of Pharmacy, The First Affiliated Hospital of Zhengzhou University, Zhengzhou, Henan 450052, P.R. China

**^3^**Affiliated Hospital of Integrated Traditional Chinese and Western Medicine, Nanjing University of Chinese Medicine, Nanjing, 210028, Jiangsu, China

**^4^**Department of Radiology, Dushu Lake Hospital Affiliated to Soochow University, Medical Center of Soochow University, Suzhou Dushu Lake Hospital, Suzhou, Jiangsu, 215123, China

Running title: Exosomal ADAM17 promotes CRC hematogenous metastasis

# These authors contributed equally to this work as co-first authors

*****Corresponding author**s**: Jinbing Sun and Yang Yang

E-mail: sunjb515@163.com (Jinbing Sun); young1570@126.com (Yang Yang)

Institute: Department of General Surgery, Changshu No. 1 People's Hospital Affiliated to Soochow University, Changshu, Jiangsu 215500, China

Mailing address: No. 1 Shuyuan Street, Changshu, Jiangsu Province, 215500, China.

Tel: 86-0512-52772228

Fax: 86-0512-52772228

**Supplementary Figures
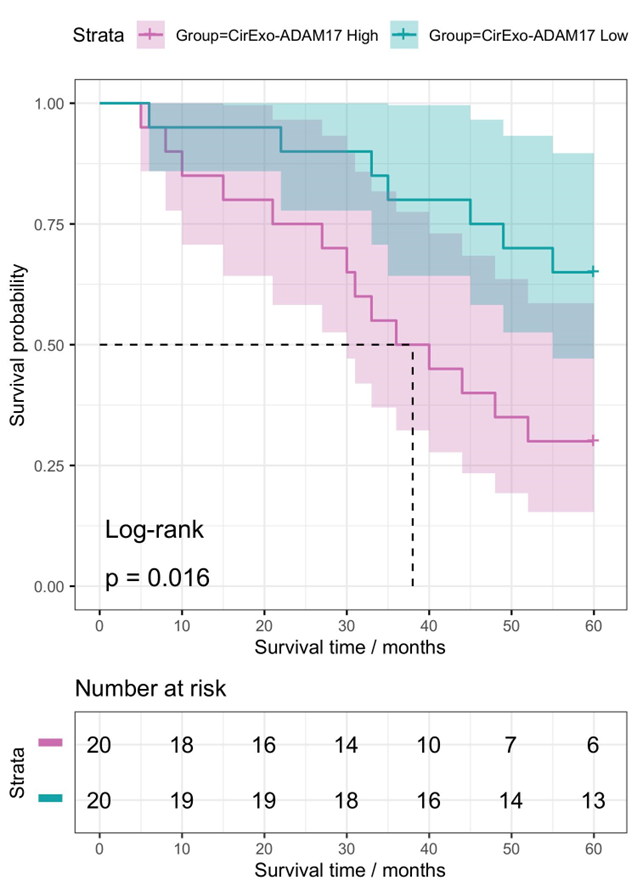
**

**Fig. S1** Univariate Kaplan–Meier analysis of the prognostic factor CirExo-ADAM17 in patients with colorectal cancer.

**
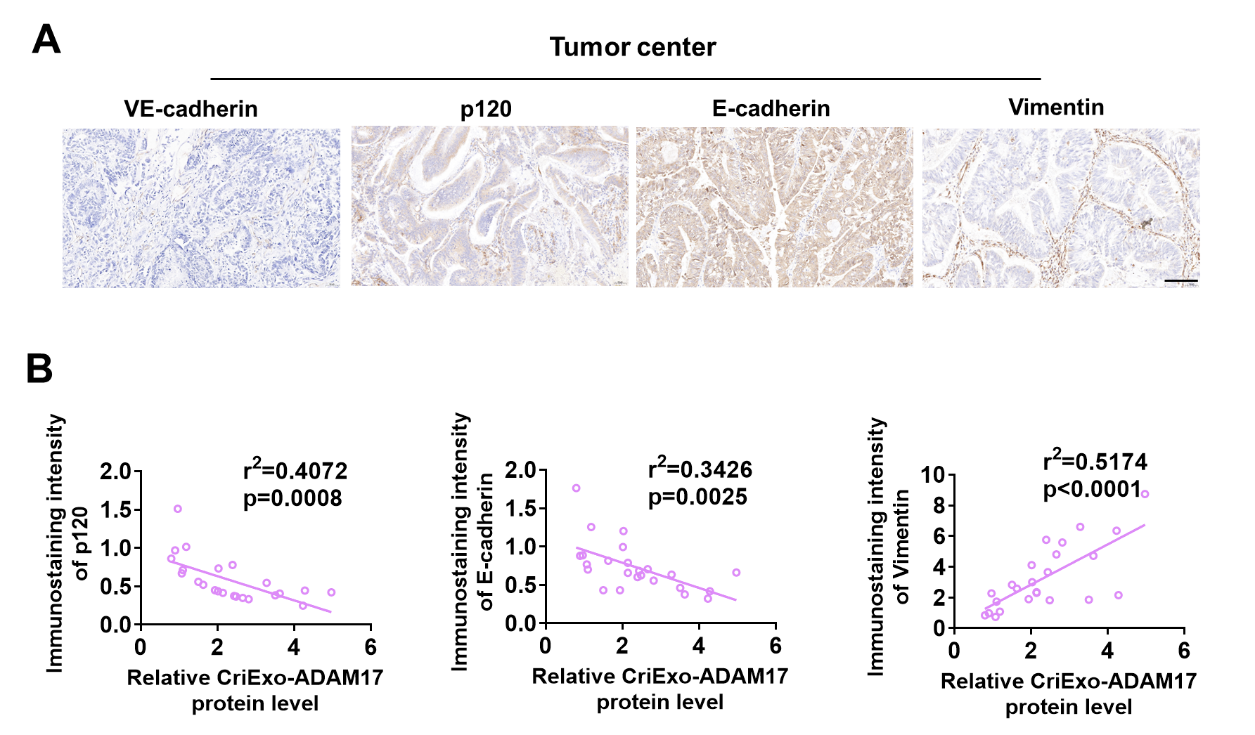
**

**Fig. S2** Correlation analysis between serum exosomal ADAM17 and colorectal cancer (CRC) metastatic protein expression. (a) Representative immunohistochemical staining for VE-cadherin, p120, and EMT (E-cadherin and vimentin) in the center of tumors derived from patients with CRC (scale bar = 100 μm). (b) Linear regression between CirExo-ADAM17 protein levels and the immunostaining intensity of p120, E-cadherin, and vimentin proteins at the invasive front.

**
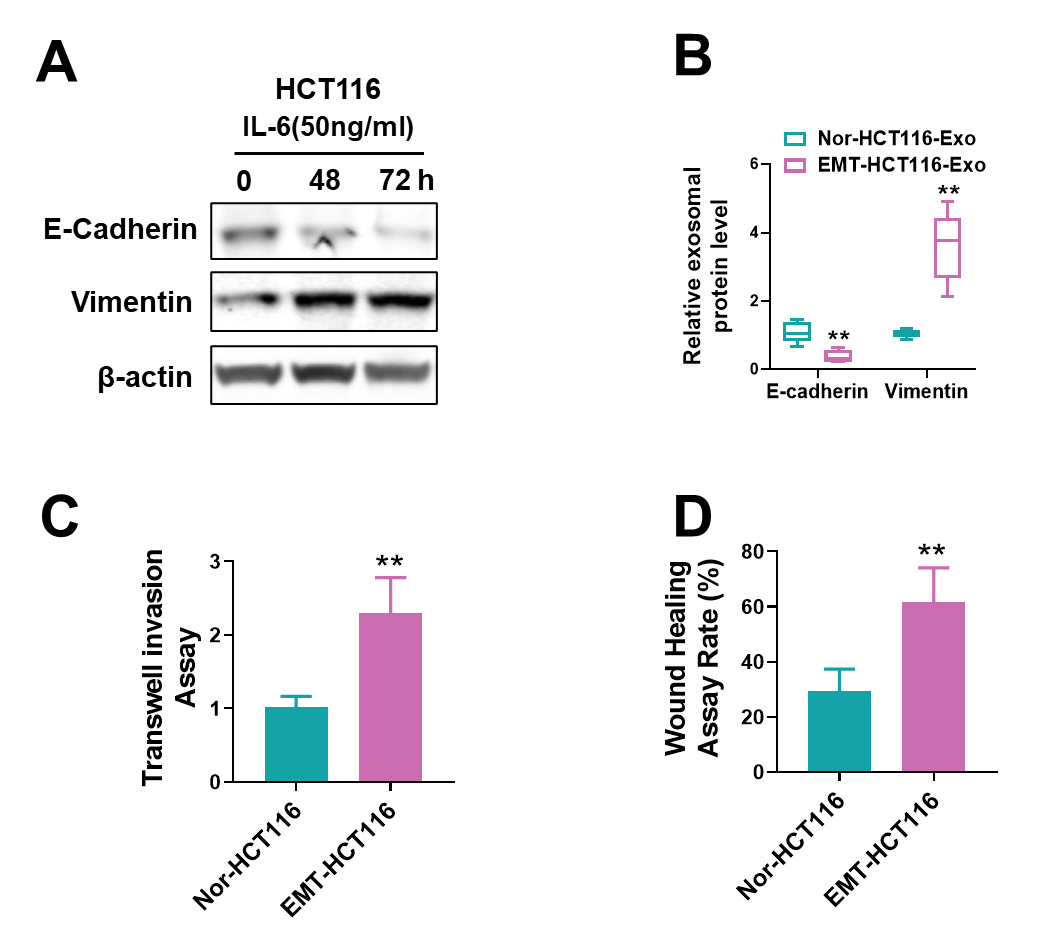
**

**Fig. S3** Transwell invasion and wound healing assays of Nor-HCT116 and EMT-HCT116 cells. (a, b) Western blotting was conducted to detect the protein expression of E-cadherin and vimentin in HCT116 cells at different time points following IL-6 treatment. (c, d) Transwell invasion assays and quantification of wound healing in Nor-HCT116 and EMT-HCT116 cells. Data are expressed as mean ± standard deviation (n = 5). *P < 0.05, **P < 0.01, compared with the Nor-HCT116 group.


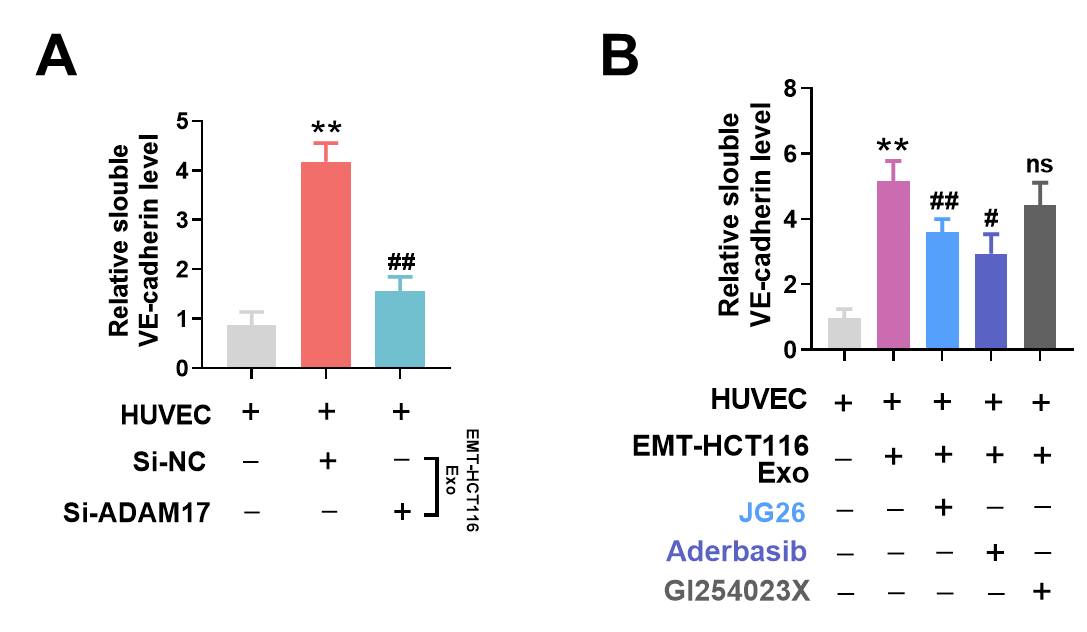


**Fig. S4** Exo-ADAM17 treatment increases the content of VE-cadherin extracellular domain fragments in the supernatant of human umbilical vein endothelial cells (HUVECs). (a) HUVECs were incubated with exosomes derived from EMT HCT116 cells (Si-NC- and Si-ADAM17-transfected), and the release of soluble VE-cadherin ectodomain was analyzed using VE-cadherin ELISA. Data are expressed as mean ± standard deviation (n = 5). **P < 0.01, compared with the control group; #P < 0.05, ##P < 0.01, compared with the Si-NC group. (b) Exosomes were pre-incubated with the ADAM17 selective inhibitor JG26 (25 nM), ADAM17 oral inhibitor aderbasib (1 μM), and ADAM10 selective inhibitor GI254023X (25 nM) for 2 h before culturing with HUVECs. The release of soluble VE-cadherin ectodomain was analyzed using VE-cadherin ELISA. Data are expressed as mean ± standard deviation (n = 5). **P < 0.01, compared with the control group; #P < 0.05, ##P < 0.01, compared with the EMT-HCT116-Exo group.

**
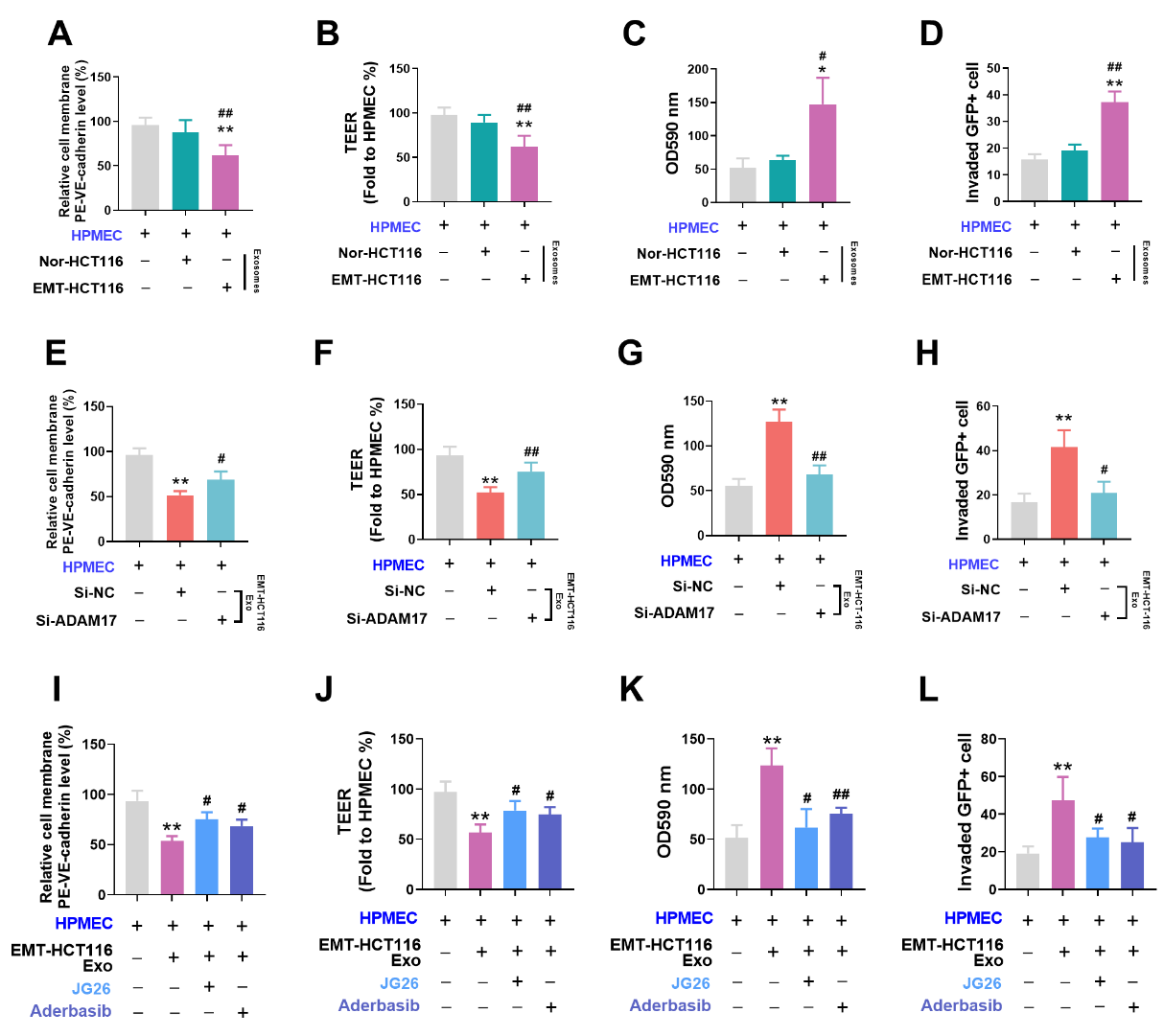
**

**Fig. S5** Exo-ADAM17 treatment increases vascular endothelial cell permeability and facilitates invasion by disrupting VE-cadherin expression in human pulmonary microvascular endothelial cells (HPMECs). (a, b) HPMEC monolayer membranes were pretreated with Nor-HCT116/EMT-HCT116-derived exosomes (50 µg; 10 μg/mL per 1 × 10^5^ cultured cells) for 4 h. Cell membrane VE-cadherin expression was measured using flow cytometry, and the TEER value was calculated. (c, d) HPMEC monolayer membranes were pretreated with Nor-HCT116/EMT-HCT116-derived exosomes (50 µg; 10 μg/mL per 1 × 10^5^ cultured cells) for 24 h; dextran content was detected (OD 590 nm) in the lower chamber and the number of tumor cells invading the HUVEC monolayer was determined. Data are expressed as the mean ± standard deviation (n = 5). *P < 0.05, **P < 0.01, compared with the control group; #P < 0.01, ##P < 0.01, compared with the Nor-HCT116 group. (e, f) HPMEC monolayer membranes were pretreated with EMT-HCT116-derived exosomes (Si-NC and Si-ADAM17 transfected) for 4 h. Cell membrane VE-cadherin expression was measured using flow cytometry, and the TEER value was calculated. (g, h) HPMEC monolayer membranes were pretreated with EMT-HCT116-derived exosomes (Si-NC and Si-ADAM17 transfected) for 24 h. Dextran content was detected (OD 590 nm) in the lower chamber and the number of tumor cells invading the HUVEC monolayer was calculated. Data are expressed as mean ± standard deviation (n = 5). *P < 0.05, **P < 0.01, compared with the control group; #P < 0.01, ##P < 0.01, compared with the Si-NC group. Exosomes were pre-incubated with ADAM17 selective inhibitor-JG26 (25 nM) and ADAM17 oral inhibitor aderbasib (1 μM) for 2 h before culturing with HUVECs. (i, j) Cell membrane VE-cadherin expression was measured using flow cytometry, and the TEER value was calculated. (k, l) Dextran content was detected (OD_590 nm_) in the lower chamber, and the number of tumor cells invading the HUVEC monolayer was determined. Data are expressed as mean ± standard deviation (n = 5). *P < 0.05, **P < 0.01, compared with the control group; #P < 0.01, ##P < 0.01, compared with the EMT-HCT116-Exo group.


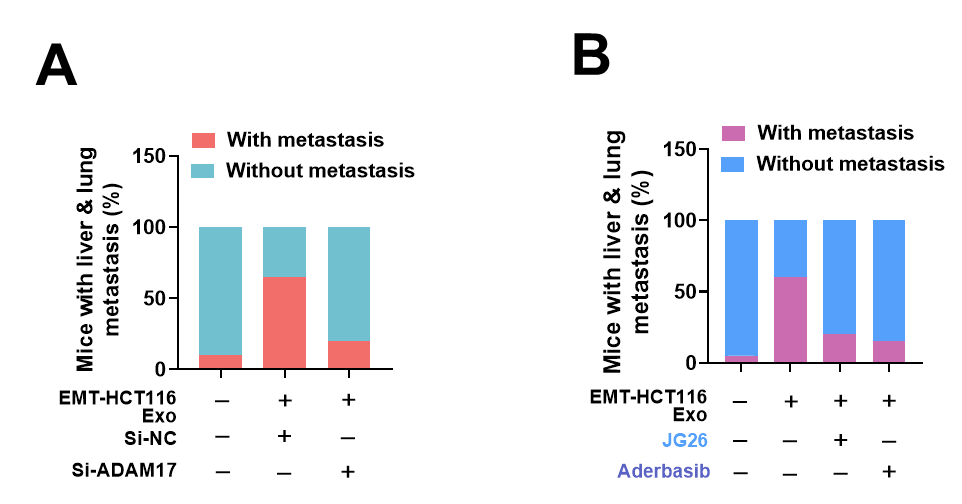


**Fig. S6** Exo-ADAM17 treatment promotes lung and liver metastases in mice with CRC. (a) Percentage of EMT- HCT116-Exo-treated mice with both lung and liver metastasis (Si-NC and Si-ADAM17 transfection). (b) Percentage of EMT-HCT116-Exo-treated mice with both lung and liver metastasis of (3 mg/kg JG26, IV; 30 mg/kg aderbasib, PO).

**Supplementary Table**

**Supplementary Table 1. cirExo-ADAM17 expression in NC (healthy donors) and patients with colorectal cancer.**

| Group | Cases, *n* | cirExo-ADAM17 expression | | P-value | χ2 |
| --- | --- | --- | --- | --- | --- |
|  |  | Positive, *n* (%) | Negative, *n* (%) |  |  |
| Colorectal cancer patients | 80 | 41 (51.25) | 39 (48.75) | P = 0.0011 | 10.73 |
| NC (healthy donors) | 30 | 5 (16.6) | 25 (83.3) |  |  |

**Supplementary Table 2.** **Differentially expressed proteins in exosomes from the non-metastatic and metastatic patients with colorectal cancer.**

| Accession | Name | Symbol |
| --- | --- | --- |
| sp\|P05121\|PAI1_HUMAN | Plasminogen activator inhibitor 1 | SERPINE1 |
| sp\|P21926\|CD9_HUMAN | CD9 antigen | CD9 |
| sp\|P07355\|ANXA2_HUMAN | Annexin A2 | ANXA2 |
| sp\|Q8WUJ3\|CEMIP_HUMAN | Cell migration-inducing and hyaluronan-binding protein | KIAA1199 |
| sp\|P08195\|4F2_HUMAN | 4F2 cell-surface antigen heavy chain | SLC3A2 |
| sp\|P78536\|ADA17_HUMAN | Disintegrin and metalloproteinase domain-containing protein 17 | ADAM17 |
| sp\|P49641\|MA2A2_HUMAN | Alpha-mannosidase 2x | MAN2A2 |
| sp\|Q14204\|DYHC1_HUMAN | Cytoplasmic dynein 1 heavy chain 1 | DYNC1H1 |
| sp\|Q99816 \|TSG101_HUMAN | Tumor Susceptibility Gene 101 Protein | TSG101 |
| sp\|Q8WUJ3\|CEMIP_HUMAN | Cell migration-inducing and hyaluronan-binding protein | CEMIP |
| sp\|Q9UL62 \|TRPC5_HUMAN | Short transient receptor potential channel 5 | TRPC5 |
| sp\|P08962\|CD63_HUMAN | CD63 antigen | CD63 |

**MATERIALS AND METHODS**

**Reagents**

JG26, aderbasib, and GI254023X were purchased from MedChem Express (Monmouth Junction, NJ, USA). Annexin V, Hoechst 33342, IL-6, and rhodamine–dextran (average MW ~70,000) were sourced from Merck (St. Louis, MO, USA). Lipofectamine 3000 transfection reagent was purchased from Thermo Fisher Scientific (Rockford, IL, USA). RIPA lysis buffer was purchased from Beyotime (Jiangsu, China). Primary antibodies used for immunoblot and immunofluorescent analyses were as follows: anti-ADAM17 (ab39163), anti-TSG101 (ab125011), anti-CD63 (ab193349), anti-TRPC5 (ab306595), anti-ANXA2 (ab189473), anti-E-cadherin (ab231303), anti-vimentin (ab92547), anti-CD34 (ab81289), anti-ZO-1 (ab307799, ab221547), FITC anti-CK (ab119107), PE-vimentin (ab49918), anti-occludin (ab216327), and anti-β-actin (ab6276) (Abcam, Cambridge, MA, USA); AF647-CD45 (E-AB-F1137M) (elabscience, USA); anti-mouse CD45.2-AF647 (1800-31) (Southernbiotech, USA); anti-Na/K-ATPase (#3010) (Cell Signaling Technology, USA); anti-VE-cadherin (sc-52751), and p120 (sc-13957) (Santa Cruz Biotechnology, Dallas, TX, USA). Goat anti-rabbit and mouse (H+L) secondary antibody, Alexa Fluor 488 and 594 conjugates, Alexa Fluor 488–conjugated goat anti-mouse IgG, Alexa Fluor 546–conjugated goat anti-rat IgG, and Alexa Fluor 647–conjugated goat anti-rat IgG were purchased from Invitrogen (Waltham, MA, USA). Goat anti-rabbit and anti-mouse IgG antibodies were purchased from LI-COR (Lincoln, NE, USA).

**CTC isolation and identification**

CTCs were enriched and identified using the CTCBIOPSY system (Wuhan YZY Medical Science and Technology, Wuhan, China). Briefly, 2.5 mL blood samples from patients with CRC were diluted into 8 mL volumes using normal saline containing 0.2% paraformaldehyde; after fixing at room temperature for 10 min, samples were transferred to ISET tubes with 8-μm diameter aperture membranes. By applying a positive pressure in the range of 12–20 mmHg, the candidate CTCs adhered to the aperture membrane; these cells were stained using three-color immunofluorescence staining; CK^+^/vimentin^-^/CD45^-^/Hoechst^+^ cells, CK^-^/vimentin^+^/CD45^-^/Hoechst^+^ cells, and CK^-^/vimentin^-^/CD45^-^/Hoechst^+^ cells were defined as epithelial CTCs, mesenchymal CTCs (MCTCs), and white blood cells, respectively. In the present study, the MCTC ratio refers to the ratio of the number of MCTCs to the total number of CTCs in 2.5 mL of peripheral blood per patient [1, 2].

**ELISA**

Quantification of soluble VE-cadherin was performed in triplicates using a VE-cadherin ELISA kit (Abcam, Cambridge, MA, USA) according to the manufacturer’s instructions.

**Wound healing assay**

For wound healing assays, cells were cultured to 90–100% confluence in 6-well plates; then, wounds were produced by scratching with a plastic pipette tip. After washing the remaining cells three times in PBS to remove cellular debris, they were incubated at 37 °C in a serum-free medium. Migrating cells at the wound front were photographed after 24 h. All experiments were performed in triplicate. Finally, wound width was determined using the ImageJ software; the percentage of wound healing was calculated according to the following formula: 100% – (width at 24 h / width at 0 h) × 100%.

**References**

1. Dou R, et al. EMT-cancer cells-derived exosomal miR-27b-3p promotes circulating tumour cells-mediated metastasis by modulating vascular permeability in colorectal cancer. Clinical and translational medicine. 2021;11(12):e595.

2. Wei C, et al. Crosstalk between cancer cells and tumor associated macrophages is required for mesenchymal circulating tumor cell-mediated colorectal cancer metastasis. Mol Cancer. 2019;18(1):64.
